# Relationship Between Health Service Use and Health Information Technology Use Among Older Adults: Analysis of the US National Health Interview Survey

**DOI:** 10.2196/jmir.1753

**Published:** 2011-04-20

**Authors:** Namkee Choi

**Affiliations:** ^1^University of Texas at AustinSchool of Social WorkAustin, TXUnited States

**Keywords:** Health information technology, older adults, health service use, Internet use

## Abstract

**Background:**

Older adults are the most frequent and heaviest users of health services in the United States; however, previous research on older adults’ use of health information technology (HIT) has not examined the possible association of HIT use among older adults with their use of health services.

**Objective:**

This study examined the relationship between US older adults’ use of health services and their use of the Internet for health-related activities, controlling for socioeconomic characteristics and aging-related limitations in sensory and cognitive function. It also examined gender differences in the pattern of association between the types of health services used and HIT use.

**Methods:**

The data for this study were drawn from the 2009 US National Health Interview Survey (NHIS), which was the first nationally representative household survey to collect data on HIT (Internet) use. First, the rates of lifetime and 12-month HIT use among sample adults (n = 27,731) by age group (18-29 to 85 and over) were analyzed. Second, bivariate analysis of sociodemographic characteristics, health status, and health service use by HIT use status among those aged 65 or older (n = 5294) was conducted. Finally, multivariate binary logistic regression analysis was used to test the study hypotheses with 12-month HIT use as the dependent variable and 12-month health service uses among the age group 65 or older as possible correlates.

**Results:**

The rates of HIT use were significantly lower among the age groups 65 or older compared with the younger age groups, although the age group 55 to 64 was not different from those younger. The rates of HIT use decreased from 32.2% in the age group 65 to 74 to 14.5% in the age group 75 to 84 and 4.9% in the 85 and older age group. For both genders, having seen or talked to a general practitioner increased the odds of HIT use. However, having seen or talked to a medical specialist, eye doctor, or physical therapist/occupational therapist (PT/OT) were significantly associated with HIT use only for older women, while having seen or talked to a mental health professional only marginally increased the odds of HIT use only for older men. Having visited or talked to a chiropractor and having had overnight hospitalization, surgery, and/or homecare services were not associated with the odds of HIT use for either gender.

**Conclusions:**

Older-adult users of general health services were more likely to use HIT than nonusers of general health services, while older-adult users of specialized health services were not different from nonusers of specialized health services in their odds of HIT use. The findings have implications for narrowing the age-related and socioeconomic status-related gaps in HIT use. The access gaps among racial/ethnic minority older adults and poorly educated and/or low-income older adults are especially striking and call for concerted efforts to facilitate Internet access and HIT use among these disadvantaged older adults.

## Introduction

An increasing number of Americans use the Internet to search for health information and engage in other health-related activities such as participating in Internet chat groups, filling and refilling prescriptions, and using email to communicate with their healthcare providers [1,2]. However, studies have consistently found significant age group differences in the rates and frequencies of Internet use for health-related activities, with older adults (aged 65 and over) lagging behind younger ones [3-5]. Older adults, in general, are less likely than younger adults to use Internet technology and engage in Internet activities such as email, social networking, and accessing information such as news and weather. Age-related disparity in computer ownership, digital subscriber line, cable, or satellite Internet connections from home or work, levels of education and income, and levels of literacy and health literacy are likely to be contributing factors to the age disparity in the use of Internet technology for accessing health information and engaging in other health-related activities [6-8].

Among older adults, socioeconomic factors also have been found to be associated with Internet use versus nonuse. A study of older adults (aged 55-74) in Spain found that although Internet users appeared to have better self-rated health than nonusers, this relationship disappeared once social class (derived from the cross-classification of occupation and educational attainment of the family’s primary income earner) was entered in the regression model [9]. In a US national public opinion survey, no African American or Hispanic American older adult in the sample reported going online for health information [4]. Other possible barriers to older adults’ using the Internet for health-related activities include factors related to the aging process itself. One study found that the oldest adults (ages 85 and over) had negative reactions to using health information webpages that lacked the design accommodations for older adults recommended by the US National Institute on Aging and the National Library of Medicine [10]. This finding implies that for some older adults, visual and other sensory impairment and slowing information-processing capacity may be barriers to seeking health information online. Another study of the role of Internet knowledge and cognitive abilities in Web-based information seeking found that older adults (aged 60 and over) performed at a lower level than younger ones (aged 18-39) only when search problems were complex, a finding that also implies that older adults with age-related cognitive deficits may face barriers to using health information technology (HIT) [11].

Older adults are the most frequent and heaviest users of US health services, including visits to general practitioners, medical specialists, emergency departments, ambulatory surgeries, inpatient hospitalizations, and home health care [12]. However, previous research on older adults’ use of HIT has not examined the possible association of their use of HIT with their use of health services. A 2001 survey of a representative adult sample of the US population found that more than 90% of the Internet health information seekers reported no impact of their Internet use on their numbers of visits to and telephone contacts with their physicians [1]. However, another national survey, conducted in 2003, found that 55% of Internet health information seekers contacted a health care professional because of information they had found online [13]. And those who had searched health information for a specific personal or loved one’s health or medical condition were significantly more likely to contact a health care provider following their search than were seekers of information unrelated to a specific personal or loved one’s health or medical condition. On the other hand, the same study also found that those who reported that they used Internet health information because it was free or because seeing a health professional was expensive were 90% less likely to contact a health care professional because of information found online than were those who did not mention cost factors [13]. Another study of Internet health information seeking among the chronically ill found that about 8% sought care from different doctors or providers than the ones they had been seeing because of the Internet information they had found and that about 30% used the Internet information to improve self-management of their conditions [14].

Although the samples that these studies used included older adults, the studies did not examine any age-specific pattern of association between health service use and HIT use. The purpose of the present study was to examine the relationship between US older adults’ use of health services and their use of the Internet for health-related activities. When socioeconomic characteristics and aging-related limitations in sensory and cognitive function are controlled for, older adults who use health services may be more likely to have engaged in online health-related activities than their peers who do not use such services for the following reasons: (1) they are likely to have greater needs for health care information in order to manage their acute or chronic medical conditions; (2) they may want sources of health care information to supplement and enhance information and knowledge they obtain from their health care providers; and (3) they are more likely to be put in situations where they have to engage in certain online health-related activities (eg, filling or refilling prescriptions, scheduling medical appointments, and emailing their health care providers).

Specifically, this study examined the question of whether specific types of health services are more likely than others to be associated with older adults’ HIT use and whether the relationship patterns differ by gender. Older adults rely heavily on their primary care physicians, who tend to be general practitioners, to deal with a variety of physical and mental health care needs ranging from preventive checkups and treatment to specialist referrals. Those who talked with or visited a general practitioner may be more likely than those who did not to have used HIT, because their talking with or visiting the doctor may indicate that they were having health problems and/or that they had a high level of health consciousness [15]. The findings of previous studies also suggest that those with chronic medical conditions and other serious illnesses (eg, cancer) may be more likely than others to search online health information [2,14,16]. Thus, it is possible that older adults who saw or visited a medical specialist or eye doctor or used such health services as inpatient hospitalization, surgery, physical therapy/occupational therapy (PT/OT), and home care may be more likely to have used HIT than their peers who did not use these health services.

Previous studies also showed that adults with mental health problems and other stigmatizing health conditions (eg, urinary incontinence or sexually transmitted diseases) were more likely to turn to the Internet for health information and communicate with a health care provider online [17,18]. One study also found that using the Internet increased health care use among those with psychiatric conditions [18]. Thus, older adults with mental health conditions may also be more likely to use online health information than discuss these conditions with those in their support network, or they may have visited mental health professionals after their online search for information about their mental health problems. Gender differences also needed to be examined, given the findings that women, including older women, use the Internet and HIT more than men [2,5,9,14].

This study tested the following hypotheses: Controlling for demographic and socioeconomic factors, self-reported sensory and memory limitations, and self-rated health, (1) older adults who had visited or talked to a general practitioner in the preceding 12 months compared with their peers who had not done so were more likely to have used HIT during the same period; (2) older adults who had visited or talked to a medical specialist or had used other health services (ie, eye doctor, PT/OT, chiropractor, inpatient hospitalization, surgery, and homecare) in the preceding 12 months were more likely to have used HIT during the same period than their peers who had not used these health services; (3) older adults who had visited or talked to a mental health professional in the preceding 12 months were more likely than their peers who had not done so to have used HIT during the same period; and (4) the pattern of association between the types of health services used and HIT use were likely to vary by gender. Although higher HIT use among women may be associated with their higher health service use, a directional hypothesis regarding the relationship between their HIT use and specific types of health services was not posited for lack of previous empirical data.

## Methods

### Data Source and Sample

The data for this study were drawn from the 2009 US National Health Interview Survey (NHIS) conducted by the National Center for Health Statistics (NCHS). The NHIS, conducted annually since 1957, is designed to collect data on the health of nationally representative samples. The survey employs a multistage sample designed to represent the civilian, noninstitutionalized population of the United States. The interviewed sample for 2009 consisted of 33,856 households, which yielded 88,446 persons in 34,460 families. Of the 88,446 persons, 27,731 persons aged 18 or older were designated “sample adults” and were asked some additional questions. In 2009, the NHIS was to our knowledge the first nationally representative household survey to collect data on Internet use of health information and medical communication. The 10 questions asked of the sample adults were fielded in the HIT supplement [19].

The present study used the public-use data file for all 27,731 sample adults to describe the rates of Internet use of health information and medical communication among different age groups. Then the focus was on the sample adults who were aged 65 or older to examine the relationship between health service use and HIT use (ie, Internet use of health information and medical communication). Of the 5493 sample adults aged 65 or older, responses from 33 individuals who were not non-Hispanic white, non-Hispanic black, Hispanic, or Asian were excluded from the analysis along with an additional 166 whose responses were answered by proxy, resulting in the final analysis sample size of 5294 adults aged 65 or older.

### Measures

#### Health Information Technology Use

Of 10 questions in the HIT supplement, 5 focused on Internet use for the following activities without a specific time frame. These were: (1) Have you ever looked up health information on the Internet? (2) Have you ever used chat groups to learn about health topics? (3) Have you ever refilled a prescription on the Internet? (4) Have you ever scheduled a medical appointment on the Internet? (5) Have you ever communicated with a healthcare provider by email? The other 5 questions focused on the respondent’s Internet use for the same activities during the preceding 12 months (eg, Did you look up health information on the Internet in the past 12 months?). Summary measures of lifetime and 12-month HIT use represented engagement (coded 1) or no engagement (coded 0) in any of the 5 activities.

#### Health Service Use

The following nine types of health services used in the preceding 12 months were selected to be included in the analysis as they represent a wide range of health services that a significant proportion of older adults use: (1) saw or talked to a general practitioner, (2) saw or talked to a medical specialist, (3) saw or talked to an eye doctor, (4) saw or talked to a PT/OT, (5) saw or talked to a chiropractor, (6) was hospitalized overnight, (7) had any surgery, (8) used homecare services, and (9) saw or talked to a mental health professional.

#### Demographic, Socioeconomic, and Health Status Covariates

Demographic, socioeconomic, and health status covariates were gender, age, race/ethnicity (non-Hispanic black, Hispanic, Asian, and non-Hispanic white, the reference group); marital status (widowed, divorced/separated, never married, and married, the reference group); level of education (less than high school, general equivalency diploma (GED) or high school diploma, some college or associate’s degree, bachelor’s degree, and master’s or doctoral degree, the reference group); family income-to-needs ratio (less than 1, 1-1.99, 2-3.99; missing, and 4 or higher, the reference group); paid work status (worked in the preceding 12 months vs did not work); any self-reported activity limitations due to a vision or hearing problem (yes vs no); any self-reported limitations due to difficulty remembering (yes vs no); and self-ratings of health (rated on a 5-point scale from 1, poor to 5, excellent). The latter was treated as a ratio-level variable.

### Analysis Strategy

First, the rates of lifetime and 12-month HIT use by age group (18-29, 30-44, 45-54, 55-64, 65-74, 75-84, and 85 and over) in each of 5 activity areas as well as in summary measures were presented. Second, bivariate analysis of sociodemographic characteristics, health status, and health service use by HIT use status among those age 65 or older were presented. Finally, to test the study hypotheses, multivariate binary logistic regression analysis—for both genders and then separately for men and women—was done with the summary measure of 12-month HIT use as the dependent variable and the 12-month health service uses among the 65 and over age group as possible correlates. The respondents with missing information as to their level of education (n = 37) were excluded from the multivariate analysis. Because of the cross-sectional nature of the data, the relationships examined were correlational, not causal. Analyses were conducted with svy commands in Stata 11 (StataCorp LP, College Station, Texas, USA) to account for the NHIS’s complex multistage sampling design.

## Results

### Health Information Technology Use in Different Age Groups

Table 1 shows that almost 51% of all adults reported that they had ever looked up health information on the Internet, but only 3.4% to 7% of them reported that they had ever used HIT for other health-related activities. Thus, the summary measure of lifetime HIT use—52.3% among all adults—appears to reflect that adults mostly had searched health information on the Internet but had not used it for other health-related activities. In the preceding 12 months, about 45% of all adults reported that they had looked up health information on the Internet, but only 3.2% to 5.9% of them reported that they had used HIT for other health-related activities. However, these average rates for all adults mask significant differences by age group, especially the differences in use between individuals in the age groups under 65 and the age groups 65 and over. For both lifetime and 12-month HIT use, the rates were significantly lower among individuals in the age groups over 65 than among individuals in the younger age groups. For example, more than half of those in the age groups under 55 and nearly half of those in the age group 55 to 64 had used HIT in the preceding 12 months compared with less than one third of those in the age group 65 to 74, less than one sixth of those in the age group 75 to 84, and less than one twentieth of the age group 85 and over. Gender differences or lack thereof by age group are also informative. In groups younger than age 65, the unadjusted rates of HIT use were significantly higher among women than among men, while in the age group 65 to 74, the rates were virtually the same. In the age groups 75 to 84 and 85 and over, the rates were significantly lower among women than men.

**Table 1:** Weighted percentage of persons who used health information technology by age group

		All	Age Group						
Use of Health Information Technology			18-29	30-44	45-54	55-64	65-74	75-84	85+
N=		27,731	5457	7087	5084	4360	2989	2002	752
**Have ever (%)**									
	Looked up health information on the Internet	50.8	59.9	60.4	55.6	52.5	35.7	16.3	5.7
	Used chat groups to learn about health topics	4.2	5.0	5.7	4.2	3.6	2.8	1.1	0.3
	Refilled prescription on Internet	7.0	4.0	7.1	8.7	10.6	8.2	3.7	0.4
	Scheduled medical appointment on Internet	3.4	3.9	4.5	4.0	3.2	1.6	0.8	0.3
	Communicated with health care provider by email	5.8	4.4	7.4	7.5	6.8	4.2	1.5	0.4
**Done any of the above (%)**		52.3	61.3	61.8	57.1	54.2	37.2	17.4	6.1
	Male^a^	47.6	53.8	52.9	49.7	49.6	37.0	20.5	11.3
	Female^a^	56.1	68.3	69.5	63.6	58.1	37.4	15.6	3.6
**In the preceding 12 months (%)**									
	Looked up health information on the Internet	44.8	53.3	54.1	48.4	46.4	30.3	13.3	4.5
	Used chat groups to learn about health topics	3.2	4.0	4.5	3.3	2.7	1.9	0.9	0.1
	Refilled prescription on Internet	5.9	3.3	5.5	7.4	9.5	7.2	3.3	0.4
	Scheduled medical appointment on the Internet	2.6	2.9	3.4	3.2	2.5	1.3	0.7	0.1
	Communicated with health care provider by email	5.8	4.4	7.4	7.5	6.8	4.2	1.5	0.4
**Done any of the above (%)**		46.5	54.7	55.6	50.2	48.8	32.2	14.5	4.9
	Male^a^	41.4	46.4	46.0	42.7	44.3	32.2	17.5	9.3
	Female^a^	50.7	62.5	64.0	56.8	52.5	32.3	12.8	2.8

^a^Gender difference in each age group, except the 65-74 group, is significant at *P* < .01.

### Sample Characteristics Among Persons Aged 65 and Over by 12-Month HIT Use Status

Table 2 shows that older adults who used HIT were significantly different from their age peers who did not do so in terms of demographic, socioeconomic, and health characteristics, and in terms of the rates of utilization of all nine types of health services. As compared with nonusers, the users included higher proportions of men, persons in the age group 65 to 74, non-Hispanic whites, married persons, and those who had worked in the preceding 12 months but included lower proportions of persons without college education and with lower income (ie, income-to-needs ratio < 2). A significantly lower proportion of users than nonusers reported limitations due to sensory or memory problems, and self-rated health was higher among users than nonusers. Despite their higher self-ratings of health, a higher proportion of users had seen or talked to a general practitioner, medical specialist, eye doctor, PT/OT, chiropractor, and/or mental health professional, or had had surgery, but a lower proportion of users had had an overnight hospitalization or had used homecare services.

**Table 2:** Sample characteristics among those 65 and over by HIT use status in the preceding 12 months: Weighted statistics

Sociodemographic and health characteristics		All	Did Not Use HIT	Used HIT	*P* Value^a^
		N=5294 (100%)	N=4078 (77%)	N=1215 (23%)	
**Gender**					< .001
	Male	38.9	37.6	43.3	
	Female	61.1	62.4	56.7	
**Age group**					< .001
	65-74	52.9	46.3	74.7	
	75-84	34.4	37.9	22.7	
	85+	12.7	15.7	2.6	
**Race/ethnicity**					< .001
	Non-Hispanic white	81.1	78.5	89.8	
	Non-Hispanic black	9.6	10.9	5.2	
	Hispanic	6.3	7.5	2.3	
	Non-Hispanic Asian	3.0	3.1	2.7	
**Marital status**					< .001
	Married/cohabiting	43.6	38.5	60.5	
	Widowed	36.7	41.8	19.5	
	Divorced/separated	14.8	14.5	16.0	
	Never married	4.9	5.2	4.0	
**Education**					< .001
	< High school	22.4	27.9	4.0	
	High school diploma or GED	32.2	35.4	21.3	
	Some college or associate’s degree	23.8	21.7	31.0	
	Bachelor’s degree	12.5	9.5	22.6	
	Some graduate school, MA/MS/PhD degree	8.5	4.8	20.9	
	Missing	0.6	0.7	0.2	
**Family income-to-need ratio**					< .001
	<1	10.1	12.4	2.4	
	1-1.99	19.4	22.1	10.3	
	2-3.99	26.4	25.8	28.2	
	4+	22.4	16.2	43.1	
	Missing	21.8	23.5	16.0	
% worked in the preceding 12 months		19.3	15.6	31.7	< .001
% reporting limitation due to hearing/vision problem		4.2	4.7	2.3	< .001
% reporting limitation due to memory impairment		6.9	7.8	3.9	< .001
Self-ratings of health (1=poor, 5=excellent), mean (SD)		3.31 (1.09)	3.21 (1.10)	3.63 (1.09)	< .001

**Health care service use in 12 months**					
	% saw or talked to a general practitioner	85.8	84.7	89.5	< .001
	% saw or talked to a medical specialist	44.9	41.8	55.0	< .001
	% saw or talked to an eye doctor	57.3	55.0	65.0	< .001
	% saw or talked to a PT/OT	14.0	12.4	19.3	< .001
	% saw or talked to a chiropractor	8.7	7.9	11.4	< .001
	% had overnight hospitalization	17.1	17.8	14.9	0.019
	% had any surgery	18.6	17.2	23.3	< .001
	% used homecare services	7.5	8.4	4.6	< .001
	% saw or talked to a mental health professional	3.7	3.0	5.9	< .001

^a^ *P* denotes difference between nonusers and users shown from chi-square tests or independent samples *t* tests.

As expected, a majority of older adults had seen/talked to a general practitioner. Further analysis (not shown in Table 2) found that those who had not seen/talked to a general practitioner were younger than those who had done so (mean 73.74 [SD 6.55] vs mean 74.78 [SD 6.67], *P* < .001) and that they had significantly better self-ratings of health (mean 3.61 [SD 1.09] vs mean 3.26 [SD 1.08], *P* < .001) and fewer chronic illnesses (mean 0.21 [SD 0.61] vs mean 0.34 [SD 0.78], *P* < .001 when hypertension, arthritis, diabetes, heart disease, stroke, lung disease, and cancer were included). The nonusers of general practitioners’ service were also less likely to have seen/talked to other health care providers, even though they did not differ from the users in educational level, income, and Medicare coverage. Thus, it appears that those who had not seen/talked to a general practitioner had less need for health services than those who had done so. On the other hand, those who had seen/talked to a medical specialist had significantly lower self-ratings of health (mean 3.12 [SD 1.09] vs mean 3.46 [SD 1.07], *P* < .001) and more chronic illness (mean 0.43 [SD 0.89] vs mean 0.22 [SD 0.63], *P* < .001 when hypertension, arthritis, diabetes, heart disease, stroke, lung disease, and cancer were included) than those who had not seen/talked to a medical specialist. However, these two groups did not differ in age.

Further analysis (not shown in Table 2) also found that women were older than men (mean 75.07 [SD 6.71] vs mean 73.95 [SD 6.53], *P* < .001) but did not differ from them in self-ratings of health. A higher proportion of women than men had seen or talked to a general practitioner and a medical specialist, had had overnight hospitalization and surgery, or had received homecare services, while no gender differences were found in the rates of seeking/talking to an eye doctor, a PT/OT, and/or a chiropractor.

### Relationship Between Health Service Use and Health Information Technology Use

For older adults of both genders, binary logistic regression results (model likelihood ratio χ^2^_29_ = 1433.64, *P* < .001) in Table 3 show that having visited or talked to a general practitioner, medical specialist, eye doctor, PT/OT, and/or mental health specialist in the preceding 12 months increased a person’s odds of having used HIT in the same period. On the other hand, having seen or talked to a chiropractor and having had overnight hospitalization, surgery, and/or homecare services were not significantly associated with HIT use. Although use of mental health services, compared with physical health services, appears to be highly correlated with HIT use, the odds ratios indicate that visiting or talking to other health care providers (ie, general practitioner, medical specialist, eye doctor, or PT/OT) had similar odds of increased HIT use. Significant covariates were gender, age, race/ethnicity, education, income-to-needs ratio, and self-ratings of health. Female gender was associated with higher odds of HIT use, while older age, being non-Hispanic black, Hispanic, or Asian, and being unmarried were associated with decreased odds of HIT use. As opposed to holding a master’s or doctoral degree, all the other educational levels were associated with decreased odds of HIT use. With respect to family income-to-needs ratio, those with levels between 2 and 3.99 were not different from those with levels greater than 4, but lower ratios or missing categories were associated with decreased odds of HIT use. Higher self-ratings of health were associated with increasing odds of HIT use, but self-reported limitations due to sensory or memory problems were not associated with HIT use.

Gender-separate analysis found gender-neutral as well as gender-specific correlational patterns (model likelihood ratio χ^2^_28_ = 588.49, *P* < .001 for men and model likelihood ratio χ^2^_28_ = 887.96, *P* < .001 for women). For both men and women, having seen or talked to a general practitioner increased the odds of HIT use. However, having seen or talked to a medical specialist, eye doctor, or PT/OT were significantly associated with HIT use only for older women. Having visited or talked to a chiropractor or having had overnight hospitalization, surgery, and/or homecare services were not associated with the odds of HIT use for either gender. Interestingly, when gender-separate analysis was done, having seen or talked to a mental health professional only marginally (*P* = .06) increased the odds of HIT use only for older men, and it was not significantly associated with older women's HIT use.

As compared with non-Hispanic white men, non-Hispanic black and older Hispanic men, but not Asian men, had lower odds of having used HIT, while Hispanic and older Asian women, but not non-Hispanic black women, had lower odds of having used HIT. Men’s marital status was not a factor significantly associated with the odds of their HIT use, while all single women had lower odds of having used HIT than all married women. Women with bachelor’s degrees did not differ from women with master’s or doctoral degrees. With respect to family income-to-needs ratio, women with levels between 1 and 3.99 did not differ from women with ratios greater than 4 in their odds of HIT use, but those with income-to-needs ratios less than 1 or with missing income data were less likely than women with ratios greater than 4 to use HIT. For men, however, level of education and income-to-needs ratio appeared to have a linear relationship with the odds of HIT use. Work status and limitations due to sensory or memory problems were not significantly associated with either gender, and self-rating of health was a significant factor only for women.

**Table 3:** Relationship between health information technology use and health service utilization in the preceding 12 months: Logistic regression analysis results

Predictor		All (N=5256)		Men (N=2059)		Women (N=3197)	
		Odds Ratio (SE)	95% CI	Odds Ratio (SE)	95% CI	Odds Ratio (SE)	95% CI
**Gender**							
	Male	1.00	1.00				
	Female	1.24 (0.13)^c^	1.01-1.52				
	Age	0.91 (0.01)^a^	0.89-0.92	0.93 (0.01)^a^	0.91-0.96	0.89 (0.01)^a^	0.87-0.91
**Race/ethnicity**							
	Non-Hispanic white	1.00	1.00	1.00	1.00	1.00	1.00
	Non-Hispanic black	0.61 (0.12)^b^	0.42-0.89	0.49 (0.14)^c^	0.27-0.86	0.72 (0.17)	0.45-1.14
	Hispanic	0.38 (0.08)^a^	0.25-0.56	0.52 (0.15)^c^	0.30-0.92	0.29 (0.08)^a^	0.16-0.50
	Asian	0.55 (0.12)^b^	0.36-0.85	0.68 (0.23)	0.34-1.34	0.47 (0.14)^b^	0.27-0.83
**Marital status**							
	Married/cohabiting	1.00	1.00	1.00	1.00	1.00	1.00
	Widowed	0.55 (0.06)^a^	0.44-0.70	0.78 (0.17)	0.51-1.20	0.47 (0.07)^a^	0.35-0.62
	Divorced/separated	0.69 (0.09)^b^	0.53-0.89	0.73 (0.16)	0.48-1.11	0.60 (0.11)^b^	0.41-0.86
	Never married	0.45 (0.12)^b^	0.27-0.75	0.54 (0.23)	0.24-1.23	0.39 (0.11)^a^	0.23-0.68
**Education**							
	Some graduate school/MA/MS/PhD	1.00	1.00	1.00	1.00	1.00	1.00
	< High school	0.08 (0.02)^a^	0.05-0.13	0.06 (0.02)^a^	0.03-0.11	0.12 (0.04)^a^	0.06-0.22
	High school diploma or GED	0.22 (0.03)^a^	0.16-0.30	0.16 (0.04)^a^	0.10-0.25	0.31 (0.06)^a^	0.21-0.47
	Some college/associate’s degree	0.47 (0.07)^a^	0.35-0.63	0.37 (0.08)^a^	0.24-0.56	0.63 (0.13)^c^	0.42-0.94
	Bachelor’s degree	0.70 (0.12)^c^	0.49-0.99	0.50 (0.11)^b^	0.32-0.78	1.03 (0.23)	0.67-1.60
**Family income-to-need ratio**							
	4+	1.00	1.00	1.00	1.00	1.00	1.00
	<1	0.32 (0.08)^a^	0.19-0.54	0.30 (0.13)^b^	0.13-0.75	0.39 (0.12)^b^	0.21-0.72
	1-1.99	0.58 (0.09)^a^	0.44-0.78	0.45 (0.12)^b^	0.27-0.85	0.72 (0.14)	0.48-1.07
	2-3.99	0.84 (0.10)	0.67-1.07	0.57 (0.11)^b^	0.39-0.78	1.15 (0.18)	0.85-1.58
	Missing	0.58 (0.07)^a^	0.44-0.75	0.51 (0.11)^b^	0.33-0.78	0.70 (0.12)^c^	0.51-0.97
Did not work in 12 months		1.00	1.00	1.00	1.00	1.00	1.00
Worked		1.10 (0.12)	0.89-1.37	1.05 (0.18)	0.75-1.47	1.23 (0.19)	0.91-1.66
No hearing/vision problem		1.00	1.00	1.00	1.00	1.00	1.00
Hearing/vision problem		0.93 (0.27)	0.52-1.66	0.69 (0.25)	0.33-1.42	1.24 (0.47)	0.59-2.61
No memory problem		1.00	1.00	1.00	1.00	1.00	1.00
Memory problem		1.04 (0.22)	0.69-1.59	0.93 (0.33)	0.46-1.88	1.21 (0.32)	0.73-2.03
Self-ratings of health		1.16 (0.06)^b^	1.05-1.28	1.04 (0.08)	0.90-1.21	1.29 (0.09)^a^	1.12-1.49
Did not see/talk to a GP		1.00	1.00	1.00	1.00	1.00	1.00
Saw/talked to a GP		1.50 (0.20)^b^	1.15-1.97	1.53 (0.29)^c^	1.05-2.21	1.52 (0.30)^c^	1.03-2.24
Did not see/talk to a specialist		1.00	1.00	1.00	1.00	1.00	1.00
Saw/talked to a medical specialist		1.35 (0.15)^b^	1.09-1.67	1.21 (0.20)	0.87-1.68	1.48 (0.21)^b^	1.12-1.95
Did not see/talk to an eye doctor		1.00	1.00	1.00	1.00	1.00	1.00
Saw/talked to an eye doctor		1.27 (0.12)^b^	1.06-1.53	1.11 (0.15)	0.85-1.44	1.41 (0.19)^b^	1.08-1.82
Did not see/talk to a PT/OT		1.00	1.00	1.00	1.00	1.00	1.00
Saw/talked to a PT/OT		1.46 (0.18)^b^	1.14-1.87	1.20 (0.24)	0.81-1.78	1.76 (0.29)^a^	1.27-2.44
Did not see/talk to a chiropractor		1.00	1.00	1.00	1.00	1.00	1.00
Saw/talked to a chiropractor		1.02 (0.15)	0.77-1.36	0.93 (0.25)	0.55-1.57	1.10 (0.19)	0.78-1.55
Did not have hospitalization		1.00	1.00	1.00	1.00	1.00	1.00
Had overnight hospitalization		0.92 (0.16)	0.66-1.29	1.44 (0.35)	0.90-2.31	0.66 (0.15)	0.42-1.04
Did not have surgery		1.00	1.00	1.00	1.00	1.00	1.00
Had any surgery		1.21 (0.16)	0.94-1.57	1.07 (0.20)	0.74-1.55	1.29 (0.26)	0.87-1.91
Did not use home care services		1.00	1.00	1.00	1.00	1.00	1.00
Used home care services		0.76 (0.16)	0.51-1.14	0.66 (0.22)	0.35-1.27	0.85 (0.23)	0.50-1.44
Did not see/talk to a mental health professional		1.00	1.00	1.00	1.00	1.00	1.00
Saw/talked to a mental health professional		1.78 (0.43)^c^	1.10-2.88	1.93 (0.68)	0.97-3.87	1.70 (0.55)	0.90-3.22

^a^ *P* < .001

^b^ *P* < .01

^c^ *P* <.05

## Discussion

Despite many previous studies that found a significant age-related digital divide in HIT use, previous research did not extensively identify contributors and barriers to older adults’ HIT use. Given that the older adult groups are the most frequent, heaviest users of health services of all age groups, the goal of the study was to examine the relationship between their HIT use and their health service use. The analysis of the US NHIS data confirmed the findings of previous studies that the rates of HIT use were significantly lower in persons in the age group 65 and over compared with persons in younger age groups, although persons in the age group 55 to 64 were not different from those in the younger age groups. The age group difference was also conspicuous among those aged 65 and over, with the rates of HIT use decreasing from 32.2% in the age group 65 to 74 to 14.5% in the age group 75 to 84 and 4.9% in the age group 85 and over. In addition to age, other demographic and socioeconomic variables (race/ethnicity and levels of education and family income) were significant determinants of HIT use among older adults.

Multivariate logistic regression results fully support hypothesis 1 (ie, a positive association between visits/talk to a general practitioner and HIT use) but only partially support hypothesis 2 (ie, a positive association between visits/talk to other health services and HIT use) and hypothesis 3 (ie, a positive association between visits/talk to a mental health professional and HIT use). Partial support for hypotheses 2 and 3 was attributable to the findings that having visited or talked to a chiropractor and having had overnight hospitalization, surgery, and/or homecare services were not associated with the odds of HIT use, while having visited or talked to a medical specialist, eye doctor, or PT/OT was significantly associated with women’s HIT use only. Given these gender-specific patterns of association between health service use and HIT use, hypothesis 4 (ie, gender difference) was supported.

The findings imply that—controlling for demographic, socioeconomic, and health status—older adults with more general health care needs were more likely to use HIT than those with fewer general health care needs, as seeing/talking to a general practitioner was a significant correlate for both genders. On the other hand, the lack of association for both genders between HIT use and the use of overnight hospitalization, surgery, chiropractic care, and homecare appears to suggest that these more severe or specialized health care needs are not significantly associated with the odds of HIT use. The older adults who had undergone an overnight hospitalization and/or outpatient or inpatient surgery and/or had received homecare (usually following a hospitalization) were likely to have received health care information specific to their medical conditions from their health care providers, decreasing the need for online information seeking. Also, serious and/or multiple medical problems may have made it difficult for these older adults to use HIT. Some older adults have difficulty searching for complex problems online or understanding complex medical information [11,20].

Gender differences in HIT use are very interesting. The unadjusted rates of HIT use were lower among older women than older men; however, in line with the findings of previous studies that included all adult age groups [3,5,8,21], multivariate analysis results show that, when other things were equal, older women were more likely than their male counterparts to have used HIT. The finding that older women who had seen or talked to a medical specialist or eye doctor were also more likely to have used HIT than their peers who had not seen or talked to those health care providers suggests that older women may be more likely than their male counterparts to look for a wide range of online health information or engage in other health-related activities. An intriguing finding is the gender-specific association between mental health service use and HIT use. The finding that only older men who had seen or talked to a mental health professional were more likely than their peers who had not done so to have used HIT suggests two possibilities: (1) as found in previous studies [17,18], the stigma of having mental health problems may have influenced older men to a greater extent than older women to utilize HIT as a source of information or vehicle for other related activities; and (2) HIT use may have influenced older men to a greater extent than older women to use mental health services.

The study has a few limitations. First, since NHIS is a cross-sectional data set, the time order between HIT use and health service use could not be determined. As a result, only correlations, not causations, were deduced. Second, the NHIS HIT questions did not ask the respondents for whom they had searched health information and engaged in health-related activities on the Internet. Although most of those who used HIT are likely to have used it for both themselves and their loved ones, some older adults may have used it exclusively for others, such as their spouse and their relatives. Longitudinal and qualitative data will help examine the timing and the context of older adults’ use of HIT. Third, the NHIS data are self-reported, thus the reliability of some data on health service use and HIT use may be questionable, especially for those with some memory issues. Future research needs to find more objective measures to examine the relationship between health service use and HIT use.

Despite these limitations, this was one of the first studies to have examined the association between older adults’ use of HIT and their health service use. The findings have implications for narrowing the age-related and socioeconomic status–related gaps in HIT use. The access gaps among racial/ethnic minority older adults and poorly educated and/or low-income older adults are especially striking and call for concerted efforts to facilitate Internet access and HIT use among these disadvantaged older adults. Previous studies show that training classes and technical support may help (1) low-income persons use the Internet use, and (2) older adults in general who are willing to use the Internet as a general source of health information and email to communicate with their physicians [22-24]. However, the content of health information and the webpage design issues are also important considerations when attempting to accommodate low level of literacy/health literacy as well as the aging-related sensory and cognitive limitations among these older adults [8,10,11]. To increase HIT use among those with complex medical conditions, ease of comprehension also needs to be considered for all age groups [20]. Given the comparable rates of HIT use between the age group 55 to 64 and the younger age groups, the HIT use of future older adults is likely to increase. However, the digital divide between racial/ethnic minority, poorly educated, and/or low-income older adults and their non-Hispanic white, better-educated, and high-income counterparts will likely continue unless there are targeted efforts to reduce the access gaps.

## Abbreviations

GED: general equivalency diploma
HIT: health information technology
NCHS: National Center for Health Statistics
NHIS: National Health Interview Survey
PT/OT: physical therapy/occupational therapy
